# A literature review and meta-analysis of the optimal factors study of repetitive transcranial magnetic stimulation in post-infarction aphasia

**DOI:** 10.1186/s40001-023-01525-5

**Published:** 2024-01-03

**Authors:** Yang Tan, Lin-Ming Zhang, Xing-ling Liang, Guei-fei Xiong, Xuan-lin Xing, Qiu-juan Zhang, Bing-ran Zhang, Zi-bin Yang, Ming-wei Liu

**Affiliations:** 1 Department of Emergency, The First Affiliated Hospital of Kunming Medical University, 295 Xichang Road, Wuhua District, Kunming, 650032 Yunnan China; 2 Department of Neurology, The First Affiliated Hospital of Kunming Medical University, Kunming, 650032 Yunnan China; 3 Department of Orthopedics, People’s Hospital of Dali Bai Autonomous Prefecture, Dali, 671000 Yunnan China; 4 Department of Emergency , People’s Hospital of Haimen District, Nantong, 226000 Jiangsu China

**Keywords:** Repetitive transcranial magnetic stimulation, Cerebral infarction, Aphasia, Optimal dose, Treatment

## Abstract

**Background:**

The existing literature indicates that repetitive transcranial magnetic stimulation (rTMS) can potentially enhance the prognosis of poststroke aphasia (PSA). Nevertheless, these investigations did not identify the most effective parameters or settings for achieving optimal treatment outcomes. This study involved a meta-analysis aimed to identify the optimal variables for rTMS in treating post-infarction aphasia to guide the use of rTMS in rehabilitating PSA.

**Methods:**

PubMed, Embase, and Cochrane Library databases were searched from inception to May 2023, and articles were reviewed manually using subject words and free words and supplemented with references from the included literature to obtain additional relevant literature. The search terms included “poststroke aphasia” and “repetitive transcranial magnetic stimulation (rTMS)” repetitive transcranial magnetic stimulation. Additionally, a review of the reference lists of previously published systematic reviews identified through the Cochrane Database of Systematic Reviews (search terms: poststroke aphasia, rTMS; restrictions: none) and PubMed (search terms: poststroke aphasia, rTMSs; restrictions: systematic review or meta-analysis) was performed. Information from studies involving different doses of rTMS in PSA was independently screened and extracted by 2 researchers.

**Results:**

This meta-analysis included 387 participants with PSA across 18 randomized controlled trials. The results showed that the total pulse had a trend toward a significant correlation with the treatment effect (*P* = 0.088), while all other variables did not correlate significantly. When rTMS was not grouped by stimulus parameter and location, our nonlinear results showed that when the total pulses were 40,000 (standardized mean difference (SMD):1.86, 95% credible interval (CrI) 0.50 to 3.33), the pulse/session was 1000 (SMD:1.05, 95% CrI 0.55–1.57), and an RMT of 80% (SMD:1.08, 95% CrI 0.60–1.57) had the best treatment effect. When rTMS was grouped by stimulus parameters and location, our nonlinear results showed that when the total low-frequency (LF)-rTMS-right inferior frontal gyrus (RIFG) pulse was 40,000 (SMD:1.76, 95% CrI:0.36–3.29), the pulse/session was 1000 (SMD:1.06, 95% CrI:0.54–1.59). Optimal results were obtained with an RMT of 80% (SMD:1.14, 95% CrI 0.54 − 1.76).

**Conclusions:**

The optimal treatment effects of rTMS for PSA may be obtained with a total pulse of 40,000, a pulse/session of 1000, and an RMT of 80%. Further rigorous randomized controlled studies are required to substantiate the validity of these results.

**Supplementary Information:**

The online version contains supplementary material available at 10.1186/s40001-023-01525-5.

## Introduction

Aphasia is a neurological condition characterized by impaired language understanding and expression. It typically develops because of damage to the brain's language centers or associated networks [1]. Brain tumors, traumatic brain injury, and intracranial infections can induce aphasia, stroke is the main cause, and poststroke aphasia (PSA) is as high as 21–38% [2]. China has an annual incidence of over 2 million stroke cases and approximately 600,000 incidents of PSA [3]. In the United States, each year, 7 million people self-report having a stroke, and approximately 100,000 stroke patients are diagnosed with PSA [4]. Cerebral infarction is the main cause of PSA, and post-infarction aphasia accounts for 62% of PSA cases, with a 4% annual increase in the risk of developing it [2, 5]. Aphasia is associated with negative effects such as anxiety [6], depression [7], impairment in social participation [8], and reduced quality of life [9]. The financial expenses associated with providing care for individuals diagnosed with aphasia are significantly greater than those without aphasia, resulting in a substantial societal burden [10]. Therefore, language rehabilitation for aphasic patients after cerebral infarction has become an urgent problem in stroke rehabilitation.

Currently, there are three main types of aphasia rehabilitation therapies: pharmacotherapy, behavioral training, and brain neuromodulation. Pharmacological therapies are divided into western and Chinese herbal therapies. Various clinical trials have provided evidence that Western medications, including meperidine, bromocriptine, donepezil, and piracetam, as well as drugs used in Chinese medicine for wind removal, phlegm dissipation, and channel dredging (such as Jieyudan Rod and flavored Jieyudan) may enhance the language function of individuals with aphasia to varying degrees [11, 12]. However, both Chinese and Western medications are associated with adverse effects, and current pharmacological treatments combined with behavioral training, neuromodulation, and pharmacological treatment alone have limited efficacy for language rehabilitation in patients with aphasia [13, 14]. In recent years, neuromodulation techniques have received increasing attention for the treatment of PSA [15–18] because they can promote the reconstruction of functional brain subdivisions and modulate neural network reorganization to exert therapeutic effects. The two primary methods of these procedures are noninvasive and invasive brain stimulation. Invasive brain stimulation is commonly used in acupuncture therapy to expand cerebral blood vessels, increase cerebral blood flow, and improve cerebral ischemia to promote the recovery of language function by needling acupuncture points in the patient’s head [17, 18]. Repetitive transcranial magnetic stimulation (rTMS) is the most commonly used noninvasive brain stimulation technique. The principle of this technique in the treatment of aphasia differs from that of acupuncture in that it applies magnetic stimulation of different frequencies to the same cortical area through an electrically charged coil to induce depolarization or hyperpolarization of synaptic cells between neurons in the brain, which in turn affects cortical activity at the stimulation site or distant sites to promote the recovery of language function [19, 20]. Studies have confirmed that rTMS can restore homeostasis in the cerebral hemispheres and improve language function in patients with aphasia by changing stimulation frequency [20–22].

In recent years, many regional and international scholars have used low and high doses of rTMS to modulate interhemispheric interactions and promote language recovery in patients [23]. However, the optimal variables for achieving the greatest treatment effects remain unclear. Thus, to determine which rTMS settings in post-infarction aphasia resulted in the highest improvement in the rehabilitation of rTMS in the treatment of PSA, a meta-analysis was performed in the current study.

## Methods

The Preferred Reporting Items for Systematic Reviews and Meta-Analyses for Network Meta-Analyses (PRISMA-NMA) guidelines were followed during the study methodology [24]. The protocol number registered in the PROSPERO database is CRD42023437016.

### Search strategy

We conducted a systematic search in electronic databases (Appendix 1), such as Web of Science, Cochrane Central Register of Controlled Trials (CENTRAL), PsycINFO, Embase, MEDLINE, and PubMed from their inception dates to May 23, 2023, through the terms ‘repetitive transcranial magnetic stimulation (rTMS)' and Medical Subject Headings (MeSH) for the terms 'poststroke aphasia’. Further investigations involved examining the reference lists of pre-existing systematic reviews that were discovered through the Cochrane Database of Systematic Reviews and PubMed (search terms: poststroke aphasia, rTMS; limitations: systematic reviews or meta-analyses) and, respectively (poststroke aphasia, rTMS; limits: none). Ethnicity and language of the trial participants were not filtered. Before being included in the search results, Ming-wei Liu eliminated any duplicates. Ming-Wei Liu and Lin-ming Zhang independently screened the titles and abstracts of the remaining articles, adhering to the predetermined inclusion and exclusion criteria. Liang and Xiong independently screened the complete texts that satisfied the abovementioned criteria. Ming-wei Liu served as the referee for all disputes, and the specific search method is presented in the Additional file 1 (see the retrieval strategy).

### Eligibility criteria

The criteria for inclusion in the study were determined using the PICOS framework, which considers participants, interventions, comparators, outcomes, and study design [1]. In order to meet the requirements for inclusion, the research must adhere to particular guidelines on the reporting of experimental variables as follows: (a) participants were diagnosed with poststroke aphasia using standard scales (i.e., the Concise Chinese Aphasia Test, Boston Diagnostic Aphasia Examination, Aphasia Severity Rating Scale, Aachen Aphasia Test, and Aphasia Rapid Test); (b) the intervention included rTMS and advanced variants; (c) comparison of sham placebo therapy or stimulation; (d) the outcome was the total language scale, such as the aphasia quotient (AQ) of the Western Aphasia Battery and Aphasia Battery of Chinese, as well as the overall score of other scales; and (e) we included published and unpublished RCTs.

Studies were excluded based on the following criteria: (a) they had a nonrandomized design; (b) the study employed therapies that were deemed irrelevant, including invasive procedures such as deep brain stimulation. (c) Means ± standard deviation (SD) were not included in the results or if the authors did not respond to our request for data; (d) the selection of control groups was deemed inappropriate, for example, healthy participants or those involved in other effective treatments; or (c) they did not clearly describe the targeted stimulation location of rTMS, resting motor threshold, or pulses per session. Following the specified criteria for inclusion and exclusion, two reviewers, Ming-wei Liu and Lin-ming Zhang, thoroughly examined potentially pertinent publications. This examination involved assessing the titles, abstracts, and full texts of articles to determine their suitability for inclusion.

### Data extraction

Two independent examiners (Ming-wei Liu and Lin-ming Zhang) assembled pertinent publication data, including author, title, year, and journal. In addition, they collected data on the number of patients, patient characteristics including age and sex, interventions examined, and outcome measures; if the original study provided a standard error for the experimental and control groups, the SD was computed using the following formula: standard deviation (SD) = standard error (SE) × √ In situations where both values were absent, the SD was estimated using several statistical measures, such as the confidence interval, t value, quartile, range, or p values, as outlined in Sect. 7.7.3 of the Cochrane Handbook for Systematic Reviews [25]. To obtain accurate measurements, the data extraction process utilized GetData (http://getdata-graph-digitizer.com) to extract the length of the axes in pixels for calibration purposes. Subsequently, the length of the pixels from the pertinent axis to the desired data points is determined. When the procedures mentioned above failed to yield the required data, we initiated contact with the authors on at least four occasions for 6 weeks.

### Evaluation of the risk of bias

Two independent reviewers (XD and JZ) evaluated the quality of the included studies using the Cochrane Risk of Bias version 2 tool (RoB2) [26] and included five domains: selection of reported results, outcome measurement, randomization process, deviations from intended interventions, and missing outcome data. The RoB2 tool incorporates an additional domain, in conjunction with the five existing domains, to evaluate the potential for bias in cluster randomized controlled trials arising from the timing of participant identification and recruitment [27]. Each area was assessed as (1) high-risk, (2) low-risk, and (3) some concern. If all domains exhibited low risk, each study's collective risk of bias was deemed low. If any of the domains mentioned above exhibited a high level of risk, or if the assessment findings of numerous domains indicated some degree of worry, then the overall risk of bias was deemed high. Conversely, if none of the domains displayed a high risk or the assessment results of many domains did not raise any concerns, the risk of bias was considered low. Disputes were settled by establishing consensus among the reviewers or by involving a third reviewer in the consultation process.

### Data synthesis

The rTMS-specific variables included the following: targeted stimulation location in Hz (e.g., low frequency: ≤ 1 Hz, high frequency: > 1 Hz), resting motor threshold (%), pulses/100 per session, and pulses/1000 (total, pulses/session × frequency × period). To verify the effect of these variables on the dose–response relationship of overall language ability on poststroke aphasia, we first performed a linear regression based on the R-environment 'metafor' package (V.4.2.2, www.r-project.org). In addition, we used the 'MBNMAdose' package to perform random-effects Bayesian model-based network meta-analysis (MBNMA) [28] to summarize the dose–response association between rTMS-specific variables and overall language ability. There was no indication that any of the key assumptions for network meta-analysis (i.e., connectedness of the network [29], consistency in the data, and transitivity [30, 31]) were violated. We compared the fit indices of a series of nonlinear functions [32] and finally chose restricted cubic splines to evaluate the nonlinear dose–response association. Based on the model that exhibits the highest level of conformity and biological credibility [33], we positioned three inflection points at the 10th, 50th, and 90th percentiles of treatment dosage. The assessment of the departure from linearity was conducted using the Wald test[34]. Given the variations in rating scales and outcome measure units among the included studies, a random-effects model was employed to aggregate the data. The effect size measure chosen for this analysis was the standardized mean difference (SMD), and the post-treatment score was accompanied by 95% credible interval (CrI). According to previous literature [35–52], the resting motor threshold ranged from to 80–110%, and rTMS had low frequencies of 0.5 and 1 Hz and a high frequency of 20 Hz. The pulse presessions included 384, 600, 800, 1000, 1200, and 1800 pulses.

## Results

### Features of the studies that were included

**Fig. 1:**
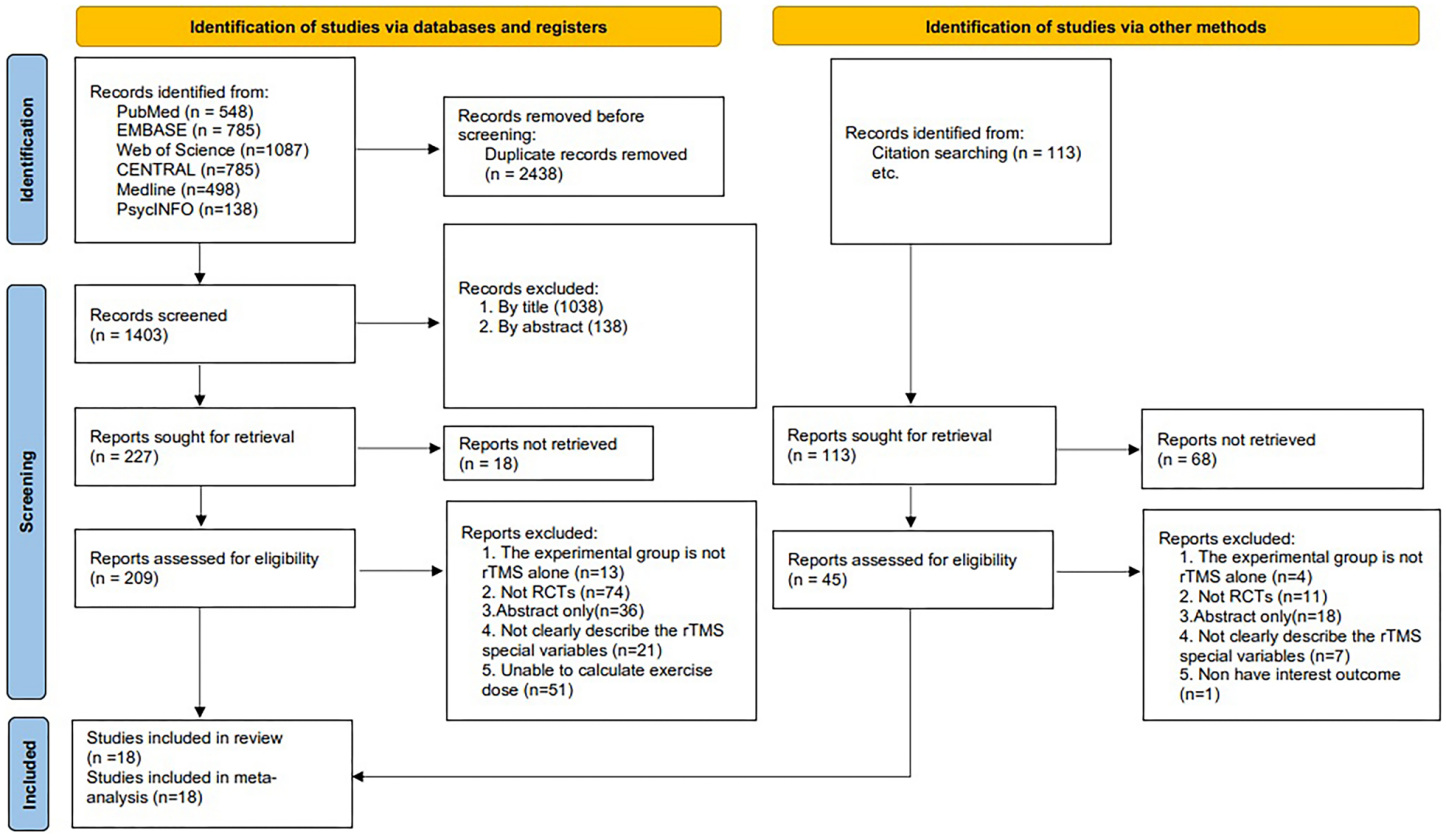
Process of literature screening

A total of 3841 studies were determined to potentially meet the eligibility criteria after an initial electronic search. Following the initial screening process of citations based on their titles and abstracts, 254 studies were selected as potentially fulfilling eligibility requirements. Subsequently, a thorough search was conducted to acquire the full-text publications of these studies. After excluding papers that did not satisfy the predetermined inclusion criteria, 18 studies [35, 36. 37. 38. 39. 40. 41. 42. 43. 44. 45. 46. 47. 48. 49. 50. 51. 52] with 387 participants (male:female 170/217) were included in the meta-analysis (Fig. 1). The sample sizes of the included studies ranged from 10 to 56. The treatment period ranged from 1 to 8 weeks, the frequency of rTMS treatment per week was 5 times, and the total sessions ranged from 5 to 40 times. The targeted stimulation locations were the right inferior frontal gyrus (RIFG), dual inferior frontal gyrus (DIFG), and right temporoparietal region (RTP) (Table 1). An assessment of the quality of the included studies is shown in Table 2.

**Table 1:** Characteristics of the included studies

Study	Mean age	Sample size (male/female)	Intervention details	Targeted brain region	Period (week)	Number of sessions	Frequency	Duration of sessions	Outcome
Bai et al. (2020)	45.3 ± 6.8	30 (13/17)	1 Hz, 80% RMT, 1000 pulses/session	RIFG	4	20/40	5/week	20 min	AQ
Barwood et al. (2013)	rTMS: 60.8 ± 6 CON: 67 ± 13.1	rTMS: 6(4/2) CON: 6(5/1)	1 Hz, 90% RMT, 1200 pulses/session	RIFG	2	10	5/week	20 min	BDAE
Chen et al. (2012)	rTMS: 62.9 ± 12.1 CON: 65.1 ± 10.9	rTMS: 22(12/10) CON: 21(11/10)	0.5 Hz, 80% RMT, 600 pulses/session	RIFG	4	20	5/week	NA	AQ
Fu et al. (2016)	rTMS: 59.77 ± 5.4 CON: 61.47 ± 6.0	rTMS: 24(14/10) CON: 24(12/12)	1 Hz, 90% RMT, 1200 pulses/session	RIFG	8	40	5/week	20 min	CRRCAE
Haghighi et al. (2017)	rTMS: 61.7 ± 7.1 CON: 60.5 ± 11.9	rTMS: 6(3/3) CON: 6(2/4)	1 Hz, 100% RMT, 1000 pulses/session	RIFG	2	10	5/week	30 min	AQ
Heiss et al. (2013)	rTMS: 68.5 ± 8.2 CON: 69.0 ± 6.3	rTMS: 17 CON: 14	1 Hz, 90% RMT, 800 pulses/session	RIFG	3–4	10	NA	20 min	AAT
Heiss et al. (2013)	rTMS: 61.0 ± 9.8 CON: 57.4 ± 9.6	rTMS: 19(8/11) CON: 10(5/5)	R: 1 Hz; L: 20 Hz; 80%-110% RMT, 1000 pulses/session	DIFG	2	10	5/week	NA	ASRS
Li et al. (2018)	rTMS: 65.3 ± 5.6 CON: 68.3 ± 5.8	rTMS: 15(9/6) CON: 15(7/8)	1 Hz, 80% RMT, 1200 pulses/session	RIFG	3	15	5/week	20 min	WAB
Ren et al. (2019)	rTMSw: 65.95 ± 8.5 rTMSb: 62.46 ± 10.9 CON: 63.6 ± 16.7	rTMS-w: 18(7/6) rTMS-b: 13(9/6) CON: 15(9/6)	1 Hz, 80% RMT, 1200 pulses/session	RIFG	3	15	5/week	20 min	AQ
Rubi-Fessen et al. (2015)	rTMS: 67.9 ± 8.1 CON: 69.6 ± 6.6	rTMS: 15(10/5) CON: 15(6/9)	1 Hz, 90% RMT, 800 pulses/session	RIFG	2	10	5/week	20 min	AAT
Seniów et al. (2013)	rTMS: 61.8 ± 11.8 CON: 59.7 ± 10.7	rTMS: 20(8/12) CON: 20(10/10)	1 Hz, 80% RMT, 1800 pulses/session	RIFG	3	15	5/week	30 min	BDAE
Shen et al. (2016)	rTMS: 60.2 ± 10.5 CON: 57.5 ± 11.9	rTMS: 20(11/9) CON: 20(8/12)	0.5 Hz, 90% RMT, 384 pulses/session	RIFG	3	15	5/week	NA	AQ
Thiel et al. (2013)	rTMS: 69.8 ± 8 CON: 71.2 ± 7.8	rTMS: 13 CON: 11	1 Hz, 90% RMT, 800 pulses/session	RIFG	2	10	5/week	20 min	AAT
Tsai et al. (2014)	rTMS: 62.3 ± 12.1 CON: 62.8 ± 14.8	rTMS: 33(24/9) CON: 23(17/6)	1 Hz, 90% RMT, 600 pulses/session	RIFG	2	10	5/week	10 min	CCAT
Waldowski et al. (2012)	rTMS: 62.3 ± 11 CON: 60.2 ± 10.6	rTMS: 13(6/7) CON: 13(7/6)	1 Hz, 90% RMT, 800 pulses/session	RIFG	3	15	5/week	30 min	BDAE-ASRS
Wang et al. (2014)	rTMS: 61.7 ± 13.8 CON: 60.4 ± 11.9	rTMS: 30(27/3) CON: 15(13/2)	1 Hz, 90% RMT, 1200 pulses/session	RIFG	2	10	5/week	20 min	CCAT
Weiduschat et al. (2011)	rTMS: 66.7 ± 9.1 CON: 63.8 ± 4.4	rTMS: 6(1/5) CON: 4(4/0)	1 Hz, 90% RMT, 1200 pulses/session	RIFG	2	10	5/week	20 min	AAT
Yoon et al. (2015)	rTMS: 60.5 ± 9.6 CON: 61.1 ± 8.7	rTMS: 10(8/2) CON: 10(7/3)	1 Hz, 90% RMT, 1200 pulses/session	RIFG	4	20	5/week	20 min	AQ

*AQ* aphasia quotient, *AAT* Aachen Aphasia Test, *BDAE* Boston Diagnostic Aphasia Examination, *CRRCA* Chinese Rehabilitation Research Center aphasia examination, *ASRS* Aphasia Severity Rating Scale, *CCAT* Concise Chinese Aphasia Test, *NA* not available, *RIFG* right inferior frontal gyrus, *DIFG* dual inferior frontal gyrus, *RTP* right temporoparietal region, *Period* period of each therapy condition

**Table 2:** Study-level risk of bias analysis

Study	Bias arising from the randomization process	Bias due to deviations from the intended intervention	Bias due to missing outcome data	Bias in measurement of the outcome	Bias in selection of the reported result	Overall
Bai et al. (2020)	Low	Low	Low	Low	Low	Low
Barwood et al. (2013)	Low	Low	Low	Low	Low	Low
Chen et al. (2012)	Low	Low	Low	Low	Low	Low
Fu et al. (2016)	Low	Low	Some concerns	Low	Low	Some concerns
Haghighi et al. (2017)	Low	Low	Low	Low	Low	Low
Heiss et al. (2013)	Low	Low	Low	Low	Low	Low
Heiss et al. (2013)	Low	Low	Low	Some concerns	Low	Some concerns
Li et al. (2018)	Low	Low	Low	Low	Low	Low
Ren et al. (2019)	Low	Low	Low	Low	Low	Low
Rubi-Fessen et al. (2015)	Low	Low	Low	Low	Low	Low
Seniów et al. (2013)	Low	Low	Low	Some concerns	Low	Some concerns
Shen et al. (2016)	Low	Low	Low	Low	Low	Some concerns
Thiel et al. (2013)	Low	Low	Low	Low	Low	Low
Tsai et al. (2014)	Low	Low	Low	Low	Low	Low
Waldowski et al. (2012)	Low	Low	Low	Low	Low	Low
Wang et al. (2014)	Low	Low	Low	Low	Low	Low
Weiduschat et al. (2011)	Low	Low	Low	Low	Low	Low
Yoon et al. (2015)	Low	Low	Low	Low	Low	Low

### Meta-analysis

**Fig. 2:**
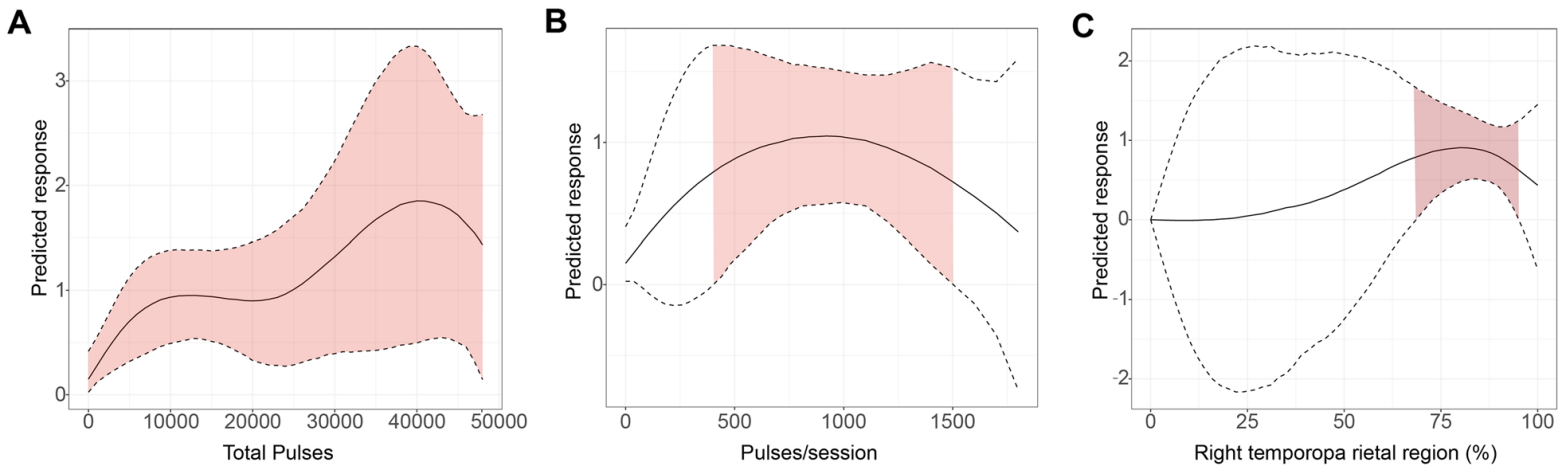
Nonlinear analysis of the effects of different total pulses (**A**), pulses/sessions (**B**), and RMT (**C**) in patients with poststroke aphasia treated with rTMS when rTMS is not grouped by stimulation parameter and location

**Fig. 3:**
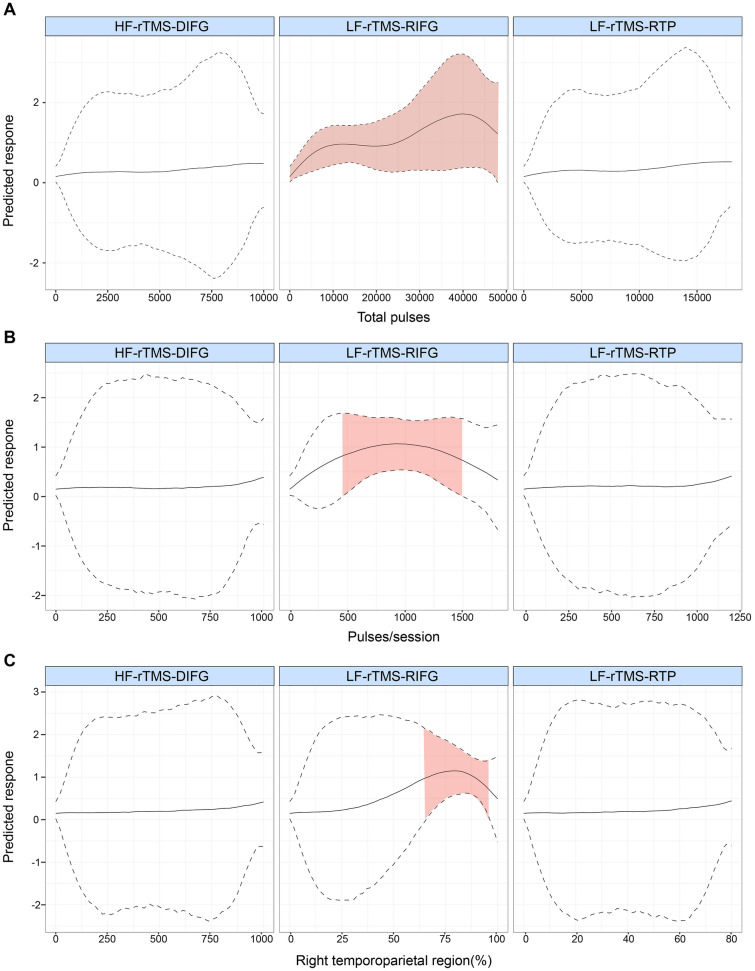
Nonlinear analysis of the effects of different total pulses (**A**), pulses/sessions (**B**), and RMT (**C**) in patients with poststroke aphasia treated with rTMS when rTMS is grouped by stimulation parameter and location

Table 3 shows the linear regression of the rTMS-specific variables for overall language ability in the PSA. Only the total pulse volume had an obvious correlation with the treatment effect (*P* = 0.088); no other variables were correlated. When rTMS was not grouped by stimulation parameter and location, our nonlinear results showed that the best therapeutic effect was observed when the total pulse was 40,000 (SMD:1.86, 95% CrI 0.50 to 3.33); pulses/session was 1000 (SMD:1.05, 95% CrI 0.55 to 1.57); and RMT was 80% (SMD:1.08, 95% CrI 0.60 to 1.57) (Fig. 2). When rTMS was grouped by stimulation parameter and location, our nonlinear results showed that the best effect was shown when the LF-rTMS-RIFG total pulse was 40000 (SMD:1.76, 95% CrI 0.36 to 3.29); pulses/session was 1000 (SMD:1.06, 95% CrI 0.54 to 1.59); and RMT was 80% (SMD:1.14, 95% CrI 0.54 to 1.76) (Fig. 3).

**Table 3:** Meta-regression results of specific stimulation variables that influence rTMS effects on total symptoms in patients with poststroke aphasia

Variables	Coefficient	Standard error	95% Lower CrI	95% Upper CrI	*P* value
Targeted stimulation location	− 0.13	0.32	− 0.79	0.54	0.696
Hz	− 0.01	0.04	− 0.09	0.07	0.748
Resting motor threshold (%)	− 0.01	0.03	− 0.07	0.05	0.662
Pulses/100 per session	− 0.02	0.05	− 0.13	0.08	0.629
Pulses/1000 (total)	0.02	0.01	− 0.04	0.05	0.088*

* trend

## Discussion

rTMS therapy is based on the theory of "hemispheric balance”, which states that under normal physiological conditions, the left and right hemispheres of the human brain are in a state of dynamic balance [21]. The dominant hemisphere of the brain governs language function; in healthy right-handed (and most left-handed) individuals, the left hemisphere is the dominant language hemisphere. Therefore, damage to different parts of the dominant hemisphere can result in various types of aphasia. Motor aphasia occurs most commonly in patients with damage to the frontal gyrus (oral expression center), whereas sensory aphasia occurs in patients with damage to the temporal gyrus (auditory comprehension center). Currently, motor aphasia is the predominant type of aphasia. Left hemisphere cortical excitability decreases in aphasic patients with damage to the dominant hemisphere, and increased excitability in the right hemisphere cortex further inhibits the left hemisphere, leading to decreased excitability in the damaged left hemisphere [22]. rTMS generates fast pulses of a certain frequency through a coil fixed onto the scalp, creating a rapidly changing magnetic field that acts on the target area and causes neuronal firing in the brain [53]. The duration of rTMS treatment is usually 10–30 min. High-frequency rTMS (HF-rTMS) (> 1 Hz) increases cortical excitability, whereas low-frequency rTMS (LF-rTMS) (≤ 1 Hz) decreases cortical excitability. rTMS has a better local therapeutic effect, and this effect persists for several months after cessation of treatment [19, 54].

A recent literature review reported that LF-rTMS and HF-rTMS may be relatively effective and safe for the treatment of PSA, with LF-rTMS playing a mainly short-term role in subacute PSA, and HF-rTMS being the most effective in improving language function in the poststroke period. More severe lesion damage in patients is associated with better HF-rTMS effects [55]. However, no studies have been performed thus far to assess variables such as total pulses, pulses/session, and the value of RMT for achieving an optimal treatment effect of rTMS on PSA[55]. This is the first study to examine this question.

Most current studies have used LF-rTMS to stimulate the undamaged hemisphere, suppress its excitability, reduce corpus callosum inhibition, and enhance the excitability of the damaged hemisphere. Many systematic evaluation studies have assessed the efficacy of LF-rTMS and yielded better results [56, 57]. For example, Sebastianelli et al. [56] evaluated whether LF-rTMS acting on the undamaged hemisphere positively affects language rehabilitation. Weiduschat et al. [57] utilized a randomized, controlled, double-blinded study design to divide 10 patients with nonfluent aphasia after subacute phase stroke into a true stimulation treatment group (six patients) and a sham stimulation control group (four patients). Stimulation of the right inferior frontal gyrus triangle was performed in the true stimulation treatment group and stimulation of unrelated brain regions at the top of the head was performed in the sham stimulation control group. The results showed that the language function of the true stimulation treatment group improved significantly after treatment compared to the pre-treatment period, with no significant improvement detected in the sham stimulation control group.

Moreover, positron emission computed tomography (PET) revealed a shift in metabolic activity to the right hemisphere during language tasks in the sham-stimulated control group but not in the true-stimulated treatment group. A randomized controlled double-anonymized trial found significant improvements in language function in 12 patients with subacute-phase aphasia who received 14 days of 1 Hz rTMS and speech training in the right hemisphere inferior frontal gyrus, with repetition and comprehension achieving moderate effects and naming achieving smaller effects [58]. Khedr et al. [59] recruited 15 patients with subacute phase aphasia and applied 1 Hz rTMS to the right subfrontal gyrus of the patients and 20 Hz rTMS to the left subfrontal gyrus and found a significant improvement in speech scores after 10 days and 2 months of intervention.

Hu et al. [23] compared the effectiveness of various rTMSfrequencies in individuals diagnosed with aphasia. The researchers randomly assigned the participants to one of four groups: high-frequency (10 Hz), low-frequency (1 Hz), sham stimulation, and control. All participants were administered a conventional treatment protocol, which included medication and frequent speech training. In the high-frequency group, stimulation was applied to the left hemisphere speech area, whereas in the low-frequency group, stimulation was targeted to the right hemisphere speech area. The results of the assessments conducted using the language scale immediately after the intervention and two months later indicated noteworthy enhancements in spontaneous speech, auditory comprehension, and aphasia quotients among participants in the low-frequency group compared to those in the high-frequency group. Nevertheless, the group exposed to high-frequency stimuli exhibited notable enhancements in repetition and aphasia quotients compared to the control group, particularly at the 2-month mark following the intervention, suggesting that LF-rTMS and HF-rTMS are beneficial for the recovery of language function in patients with aphasia, but that LF-rTMS produces both short-term and long-term benefits.

In contrast, HF-rTMS alone produces long-term benefits, and the benefits accrued through LF-rTMS appear more significant. The variables that contribute to the optimal treatment effect of LF-rTMS-RIFG, such as total pulse, pulse/session, and RMT values, have not been explored. When grouped by rTMS stimulation parameters and location, our nonlinear results showed that the best treatment effect was achieved when the total LF-rTMS-RIFG pulse was 40,000 (SMD:1.76, 95% CrI 0.36–3.29) and the pulse/session was 1000 (SMD:1.06, 95% CrI 0.54–1.59).

To study the mechanism of action of rTMS, Thiel et al. [47] conducted LF-rTMS in patients with aphasia and found that rTMS inhibited the adverse activation of the right cerebral hemisphere, leading to weakened inhibition of language-related regions of the left cerebral hemisphere and promoting the rebalancing of the bilateral cerebral hemispheres, thus improving the language function of patients with aphasia. Most studies have used unilateral hemispheric stimulation, and only a few have used bilateral hemispheric stimulation. In 2014, Khedr et al. [60] performed the first clinical study involving bilateral hemisphere stimulation, in which subjects were randomly divided into 2 groups: a bilateral hemisphere Broca's area stimulation group (experimental group) and a sham stimulation group. The results showed that the patients in the experimental group experienced significant improvements in language function compared to those in the control group. Vuk-Sanov et al. [61] divided subjects into a bilateral rTMS group and a unilateral rTMS group (control group), with bilateral rTMS being more effective in promoting the recovery of language function in patients with aphasia. In the present study, when rTMS was not grouped by stimulation parameters and location, our nonlinear results showed that the best results were obtained when the total pulse was 40,000 (SMD:1.86, 95% CrI 0.50 to 3.33), pulse/session was 1,000 (SMD:1.05, 95% CrI 0.55–1.57), and RMT was 80% (SMD:1.08, 95% CrI 0.60–1.57), which was also applied to patients with bilateral rTMS.

In 2005, Winhuise et al. [62] administered HF-rTMS at 4 Hz to the right inferior frontal gyrus of patients with aphasia after subacute left-sided cerebral infarction. The results suggest that patients treated with HF-rTMS showed higher activation in the right inferior frontal gyrus and had lower language abilities than those with aphasia who did not receive HF-rTMS when assessed for relevant language tasks. In a subsequent study, Szaflarski et al. [63] treated eight patients with chronic aphasia with an iTBS stimulation pattern in the left speech area with HF-rTMS (50 Hz) 5 days per week and observed that language function was restored. Nevertheless, recent research has indicated that HF-rTMS targeting the non-dominant hemisphere can be a viable therapeutic approach for enhancing language abilities in patients with PSA, particularly when the extent of the brain lesion is large. In another study, five patients with aphasia after massive cerebral infarction in the left cerebral hemisphere were randomized to three stimulation patterns of high-frequency (10 Hz), low-frequency (1 Hz), and sham stimulation in the right inferior frontal gyrus, each at an interval of 6 days, and were assessed using a picture-naming task that was performed immediately before and after each rTMS treatment. HF-rTMS treatment significantly improved naming ability compared with LF-rTMS and sham stimulation treatments [55].

Currently, there is a lack of research investigating the impact of total pulses, pulses per session, and RMT values on the optimal therapeutic outcome of HF rTMS for the treatment of PSA. In our study, when rTMS was not grouped by stimulation parameter and location, our nonlinear results showed that the best results were obtained when the total pulse was 40,000 (SMD:1.86, 95% CrI 0.50 to 3.33); pulse/session was 1000 (SMD:1.05, 95% CrI 0.55–1.57), and RMT was 80% (SMD:1.08, 95% CrI 0.60–1.57). It is hypothesized that these variables are also appropriate for patients with aphasia treated with HF-rTMS; however, the results must be further validated.

## Limitations and strengths

*Strengths*: This study provides evidence for selecting the optimal pulse, pulse/session, and RMT for rTMS in PSA.

*Limitations*: Case studies and clinical trials differ in sample selection (e.g., lesion size/site), stimulation pattern, frequency of stimulation, and site of stimulation, which may bias the study results. Recently, there has been a growing tendency to highlight the importance of tailored TMS and, in general, multimodal (integrating noninvasive brain stimulation with other approaches such as cognitive training and physical exercise) rehabilitation programs. In addition, the heterogeneous nature of samples with post-infarction aphasia (as the characteristics and spread of the damaged area are unique to each patient) renders the advice of tailored TMS and cognitive rehabilitation protocols even more important. As a treatment, 1000 pulses per session may be delivered by employing numerous different protocols, which would have very different effects (for instance, at a low frequency, at a high frequency, using intermittent or continuous theta bursts). Therefore, additional prospective cohort studies and randomized controlled trials are required to enhance the existing body of evidence and demonstrate a definitive causal relationship.

## Conclusions

The results of the meta-analysis of the stimulus-specific variables affecting the effect of rTMS on total symptoms in patients with PSA found that only total pulse correlated significantly with treatment outcome. LF-rTMS and HF-rTMS have been used to improve language function in patients with PSA. rTMS for PSA was most effective when the total pulse was 40,000, pulse/session was 1000, and RMT was 80%. This meta-analysis of clinical outcomes and selection of rTMS parameters for post-infarction aphasia provides a basis for evidence-based medical decisions regarding PSA. High-quality, randomized, controlled clinical studies with large sample sizes are needed to explore the stimulation parameters and sites for different stroke lesion/injury sites and aphasia types, which will improve the quality of clinical studies and provide more reliable evidence for rTMS in post-infarction aphasia.

### Supplementary Information

**Additional file 1.** Retrieval strategy.

## Data Availability

The datasets used and/or analyzed during the current study are available from the corresponding author upon reasonable request.

## Abbreviations

rTMS: Repetitive transcranial magnetic stimulation
PSA: Poststroke aphasia
CrI: Credible interval
AQ: Aphasia quotient
AAT: Aachen Aphasia Test
BDAE: Boston Diagnostic Aphasia Examination
CRRCA: The Chinese Rehabilitation Research Center aphasia examination
ASRS: Aphasia Severity Rating Scale
CCAT: The Concise Chinese Aphasia Test
RIFG: Right inferior frontal gyrus
DIFG: Dual inferior frontal gyrus
RTP: Right temporoparietal region
PRISMA-NMA: Preferred Reporting Items for Systematic Reviews and Meta-Analyses for Network Meta-Analyses
