# Supplementary material for: A literature review and meta-analysis of the optimal factors study of repetitive transcranial magnetic stimulation in post-infarction aphasia
Source: Eur J Med Res. 2024 Jan 3;29:18. doi: 10.1186/s40001-023-01525-5 (PMC10762838; doi:10.1186/s40001-023-01525-5)
Supplement: Supplementary file 1 — Additional file 1. Retrieval strategy. [file 40001_2023_1525_MOESM1_ESM.docx]

**Supplementary**

Table of Contents:

[Supplementary 1: Search Strategy 1](#_Toc143793371)

[Database: PubMed <inception to May 23 2023> 2](#_Toc143793372)

[Database: Ovid MEDLINE(R) <1946 to May 23 2023> 5](#_Toc143793372)

[Database: Embase <1974 to May 23 2023> 6](#_Toc143793372)

[Database: PsycINFO <1806 to May 23 2023> 7](#_Toc143793372)

[Cochrane 9](#_Toc143793372)

[Database: Web of Science <1965 to May 23 2023> 1](#_Toc143793372)0

# Supplementary 1: Search Strategy

## Database: PubMed <inception to May 23 2023>

***Search Strategy:***

| Search number | Query |
| --- | --- |
| 22 | (((((Transcranial Magnetic Stimulation[MeSH Terms] OR ((Magnetic Stimulation, Transcranial) OR (Magnetic Stimulations, Transcranial) OR (Stimulation, Transcranial Magnetic) OR (Stimulations, Transcranial Magneticl) OR (Transcranial Magnetic Stimulations) OR (Transcranial Magnetic Stimulation, Single Pulse) OR (Transcranial Magnetic Stimulation, Paired Pulse) OR (Transcranial Magnetic Stimulation, Repetitive) OR (Non-invasive electrical stimulation)[Title/Abstract])) AND ((((((((randomized controlled trial[Publication Type]) OR (controlled clinical trial[Publication Type])) OR (randomized[Title/Abstract])) OR (placebo[Title/Abstract])) OR (randomly[Title/Abstract])) OR (trial[Title])) OR (clinical trials as topic[MeSH Terms])) NOT ((animals[MeSH Terms]) NOT (humans[MeSH Terms])))) AND ((Srtoke[MeSH Terms]) OR ((Cerebrovascular Accident) OR (Brain Vascular Accident) OR (Apoplexy) OR (Cerebral Stroke) OR (Cerebrovascular Apoplexy))))) AND ((Aphasia[MeSH Terms]) OR ((Alogia) OR (Alogias) OR (Logasthenia) OR (Logasthenias) OR (Logagnosia) OR (Logagnosias) OR (Logamnesia) OR (Logamnesias) OR (Word Deafness) OR (Deafness, Word) OR (Anepia) OR (Anepias) OR (Aphasia, Semantic) OR (Semantic Aphasia) OR (Semantic Aphasias) OR (Aphasia, Syntactical) OR (Syntactical Aphasia) OR (Syntactical Aphasias) OR (Aphasia, Progressive) OR (Progressive Aphasia) OR (Aphasia, Acquired) OR (Acquired Aphasia) OR (Aphasia, Ageusic) OR (Ageusic Aphasia) OR (Ageusic Aphasias) OR (Aphasia, Auditory Discriminatory) OR (Auditory Discriminatory Aphasia) OR (Auditory Discriminatory Aphasias) OR (Discriminatory Aphasia, Auditory) OR (Discriminatory Aphasias, Auditory) OR (Aphasia, Commisural) OR (Aphasias, Commisural) OR (Commisural Aphasia))))) |
| 21 | (Aphasia[MeSH Terms]) OR ((Alogia) OR (Alogias) OR (Logasthenia) OR (Logasthenias) OR (Logagnosia) OR (Logagnosias) OR (Logamnesia) OR (Logamnesias) OR (Word Deafness) OR (Deafness, Word) OR (Anepia) OR (Anepias) OR (Aphasia, Semantic) OR (Semantic Aphasia) OR (Semantic Aphasias) OR (Aphasia, Syntactical) OR (Syntactical Aphasia) OR (Syntactical Aphasias) OR (Aphasia, Progressive) OR (Progressive Aphasia) OR (Aphasia, Acquired) OR (Acquired Aphasia) OR (Aphasia, Ageusic) OR (Ageusic Aphasia) OR (Ageusic Aphasias) OR (Aphasia, Auditory Discriminatory) OR (Auditory Discriminatory Aphasia) OR (Auditory Discriminatory Aphasias) OR (Discriminatory Aphasia, Auditory) OR (Discriminatory Aphasias, Auditory) OR (Aphasia, Commisural) OR (Aphasias, Commisural) OR (Commisural Aphasia)) |
| 20 | (Srtoke[MeSH Terms]) OR ((Cerebrovascular Accident) OR (Brain Vascular Accident) OR (Apoplexy) OR (Cerebral Stroke) OR (Cerebrovascular Apoplexy)) |
| 19 | (((((((randomized controlled trial[Publication Type]) OR (controlled clinical trial[Publication Type])) OR (randomized[Title/Abstract])) OR (placebo[Title/Abstract])) OR (randomly[Title/Abstract])) OR (trial[Title])) OR (clinical trials as topic[MeSH Terms])) NOT ((animals[MeSH Terms]) NOT (humans[MeSH Terms])) |
| 18 | ((((((randomized controlled trial[Publication Type]) OR (controlled clinical trial[Publication Type])) OR (randomized[Title/Abstract])) OR (placebo[Title/Abstract])) OR (randomly[Title/Abstract])) OR (trial[Title])) OR (clinical trials as topic[MeSH Terms]) |
| 17 | Transcranial Magnetic Stimulation[MeSH Terms] OR ((Magnetic Stimulation, Transcranial) OR (Magnetic Stimulations, Transcranial) OR (Stimulation, Transcranial Magnetic) OR (Stimulations, Transcranial Magneticl) OR (Transcranial Magnetic Stimulations) OR (Transcranial Magnetic Stimulation, Single Pulse) OR (Transcranial Magnetic Stimulation, Paired Pulse) OR (Transcranial Magnetic Stimulation, Repetitive) OR (Non-invasive electrical stimulation)[Title/Abstract]) |
| 16 | (animals[MeSH Terms]) NOT (humans[MeSH Terms]) |
| 15 | humans[MeSH Terms] |
| 14 | animals[MeSH Terms] |
| 13 | clinical trials as topic[MeSH Terms] |
| 12 | trial[Title] |
| 11 | randomly[Title/Abstract] |
| 10 | placebo[Title/Abstract] |
| 9 | randomized[Title/Abstract] |
| 8 | controlled clinical trial[Publication Type] |
| 7 | randomized controlled trial[Publication Type] |
| 6 | (Magnetic Stimulation, Transcranial) OR (Magnetic Stimulations, Transcranial) OR (Stimulation, Transcranial Magnetic) OR (Stimulations, Transcranial Magneticl) OR (Transcranial Magnetic Stimulations) OR (Transcranial Magnetic Stimulation, Single Pulse) OR (Transcranial Magnetic Stimulation, Paired Pulse) OR (Transcranial Magnetic Stimulation, Repetitive) OR (Non-invasive electrical stimulation)[Title/Abstract] |
| 5 | Transcranial Magnetic Stimulation[MeSH Terms] |
| 4 | (Alogia) OR (Alogias) OR (Logasthenia) OR (Logasthenias) OR (Logagnosia) OR (Logagnosias) OR (Logamnesia) OR (Logamnesias) OR (Word Deafness) OR (Deafness, Word) OR (Anepia) OR (Anepias) OR (Aphasia, Semantic) OR (Semantic Aphasia) OR (Semantic Aphasias) OR (Aphasia, Syntactical) OR (Syntactical Aphasia) OR (Syntactical Aphasias) OR (Aphasia, Progressive) OR (Progressive Aphasia) OR (Aphasia, Acquired) OR (Acquired Aphasia) OR (Aphasia, Ageusic) OR (Ageusic Aphasia) OR (Ageusic Aphasias) OR (Aphasia, Auditory Discriminatory) OR (Auditory Discriminatory Aphasia) OR (Auditory Discriminatory Aphasias) OR (Discriminatory Aphasia, Auditory) OR (Discriminatory Aphasias, Auditory) OR (Aphasia, Commisural) OR (Aphasias, Commisural) OR (Commisural Aphasia) |
| 3 | Aphasia[MeSH Terms] |
| 2 | (Cerebrovascular Accident) OR (Brain Vascular Accident) OR (Apoplexy) OR (Cerebral Stroke) OR (Cerebrovascular Apoplexy) |
| 1 | Stroke[MeSH Terms] |

## Database: Ovid MEDLINE(R) <1946 to May 23 2023>

***Search Strategy:***

1 Aphasia$.mp.

2 exp Alogia/

3 Stroke$.mp

4 exp Cerebrovascular Accident/

5 exp Brain Vascular Accident/

6 exp Apoplexy/

7 exp Cerebral Stroke/

8 exp Cerebrovascular Apoplexy/

9 (Magnetic Stimulation, Transcranial) OR (Magnetic Stimulations, Transcranial) OR (Stimulation, Transcranial Magnetic) OR (Stimulations, Transcranial Magneticl) OR (Transcranial Magnetic Stimulations) OR (Transcranial Magnetic Stimulation, Single Pulse) OR (Transcranial Magnetic Stimulation, Paired Pulse) OR (Transcranial Magnetic Stimulation, Repetitive) OR (Non-invasive electrical stimulation).mp.

10 exp Transcranial Magnetic Stimulation/

11 randomized controlled trial.pt.

12 controlled clinical trial.pt.

13 randomized.ab.

14 clinical trials as topic.sh.

15 randomly.ab.

16 trial.ti.

17 exp clinical trial/

18 exp randomized controlled trials/

19 exp cross-over studies/

20 (clinic$ adj2 trial).mp.

21 (random$ adj5 control$ adj5 trial$).mp.

22 (crossover or cross-over).mp.

23 randomi$.mp.

24 (random$ adj5 (assign$ or allocat$ or assort$ or reciev$)).mp.

25 1 or 2

26 3 or 4 or 5 or 6 or 7 or 8

27 9 or 10

28 11 or 12 or 13 or 14 or 15 or 16 or 17 or 18 or 19 or 20 or 21 or 22 or 23 or 24

29 25 and 26 and 27 and 28

## Database: Embase <1974 to May 23 2023>

***Search Strategy:***

1 Aphasia$.mp.

2 exp Alogia/

3 Stroke$.mp

4 exp Cerebrovascular Accident/

5 exp Brain Vascular Accident/

6 exp Apoplexy/

7 exp Cerebral Stroke/

8 exp Cerebrovascular Apoplexy/

9 (Magnetic Stimulation, Transcranial) OR (Magnetic Stimulations, Transcranial) OR (Stimulation, Transcranial Magnetic) OR (Stimulations, Transcranial Magneticl) OR (Transcranial Magnetic Stimulations) OR (Transcranial Magnetic Stimulation, Single Pulse) OR (Transcranial Magnetic Stimulation, Paired Pulse) OR (Transcranial Magnetic Stimulation, Repetitive) OR (Non-invasive electrical stimulation).mp.

10 exp Transcranial Magnetic Stimulation/

11 randomized controlled trial.pt.

12 controlled clinical trial.pt.

13 randomized.ab.

14 clinical trials as topic.sh.

15 randomly.ab.

16 trial.ti.

17 exp clinical trial/

18 exp randomized controlled trials/

19 exp cross-over studies/

20 (clinic$ adj2 trial).mp.

21 (random$ adj5 control$ adj5 trial$).mp.

22 (crossover or cross-over).mp.

23 randomi$.mp.

24 (random$ adj5 (assign$ or allocat$ or assort$ or reciev$)).mp.

25 1 or 2

26 3 or 4 or 5 or 6 or 7 or 8

27 9 or 10

28 11 or 12 or 13 or 14 or 15 or 16 or 17 or 18 or 19 or 20 or 21 or 22 or 23 or 24

29 25 and 26 and 27 and 28

## Database: PsycINFO <1806 to May 23 2023>

***Search Strategy:***

| S1 | Aphasia* | APA PsycInfo® |
| --- | --- | --- |
| S2 | mainsubject(Alogia) | APA PsycInfo® |
| S3 | Stroke* | APA PsycInfo® |
| S4 | mainsubject(Cerebrovascular Accident) | APA PsycInfo® |
| S5 | mainsubject(Brain Vascular Accident) | APA PsycInfo® |
| S6 | mainsubject(Apoplexy) | APA PsycInfo® |
| S7 | mainsubject(Cerebrovascular Apoplexy) | APA PsycInfo® |
| S8 | mainsubject(Cerebral Stroke) | APA PsycInfo® |
| S9 | su((Magnetic Stimulation, Transcranial) OR (Magnetic Stimulations, Transcranial) OR (Stimulation, Transcranial Magnetic) OR (Stimulations, Transcranial Magneticl) OR (Transcranial Magnetic Stimulations) OR (Transcranial Magnetic Stimulation, Single Pulse) OR (Transcranial Magnetic Stimulation, Paired Pulse) OR (Transcranial Magnetic Stimulation, Repetitive) OR (Non-invasive electrical stimulation)). | APA PsycInfo® |
| S10 | su(Transcranial Magnetic Stimulation$) | APA PsycInfo® |
| S11 | ab(randomized) | APA PsycInfo® |
| S12 | ab(randomly) | APA PsycInfo® |
| S13 | ti(trial) | APA PsycInfo® |
| S14 | ab(clinical trial) | APA PsycInfo® |
| S15 | ab(randomized controlled trials) | APA PsycInfo® |
| S16 | ab(cross-over studies) | APA PsycInfo® |
| S17 | ab(crossover studies) | APA PsycInfo® |
| S18 | ab(randomi*) | APA PsycInfo® |
| S19 | su(animals) | APA PsycInfo® |
| S20 | S1 OR S2 | APA PsycInfo® These databases are searched for part of your query. |
| S21 | S3 OR "S4" OR "S5" OR S6 OR "S7" OR "S8" | APA PsycInfo® These databases are searched for part of your query. |
| S22 | S9 OR S10 | APA PsycInfo® These databases are searched for part of your query. |
| S23 | S11 OR S12 OR "S13" OR "S14" OR "S15" OR "S16" OR S17 OR S18 | APA PsycInfo® These databases are searched for part of your query. |
| S24 | S20 AND S21 | APA PsycInfo® These databases are searched for part of your query. |
| S25 | S22 AND S23 | APA PsycInfo® These databases are searched for part of your query. |
| S26 | S24 AND S25 | APA PsycInfo® These databases are searched for part of your query. |
| S27 | S26 NOT S18 | APA PsycInfo® These databases are searched for part of your query. |

## Cochrane

#1 MeSH descriptor: [Aphasia] explode all trees

#2 MeSH descriptor: [Stroke] explode all trees

#3 (Magnetic Stimulation, Transcranial) OR (Magnetic Stimulations, Transcranial) OR (Stimulation, Transcranial Magnetic) OR (Stimulations, Transcranial Magneticl) OR (Transcranial Magnetic Stimulations) OR (Transcranial Magnetic Stimulation, Single Pulse) OR (Transcranial Magnetic Stimulation, Paired Pulse) OR (Transcranial Magnetic Stimulation, Repetitive) OR (Non-invasive electrical stimulation) in Trials (Word variations have been searched)

#4 MeSH descriptor: [Transcranial Magnetic Stimulation] explode all trees

#5 #3 or #4

#6 #1 and #2 and #5

## Database: Web of Science <1965 to May 23 2023>

| # 5 | #1 AND #2 AND #3 AND #4  Indexes=SCI-EXPANDED, SSCI, A&HCI, CPCI-S, CPCI-SSH, BKCI-S, BKCI-SSH, ESCI, CCR-EXPANDED, IC Timespan=All years |  |  |
| --- | --- | --- | --- |
| # 4 | TOPIC: ((“randomized controlled trial*” or “controlled clinical trial” or “random*” or “clinical trial*” or randomly or trial or “clinical trial” or “randomized controlled trial*” or “cross-over studies” or clinic*) )  Indexes=SCI-EXPANDED, SSCI, A&HCI, CPCI-S, CPCI-SSH, BKCI-S, BKCI-SSH, ESCI, CCR-EXPANDED, IC Timespan=All years |  |  |
| # 3 | TOPIC: (“Transcranial Magnetic Stimulation” or “Magnetic Stimulation, Transcranial” or “Magnetic Stimulations, Transcranial” or “Stimulation, Transcranial Magnetic” or “Stimulations, Transcranial Magneticl*” or “Transcranial Magnetic Stimulations” or “Transcranial Magnetic Stimulation, Single Pulse” or “Transcranial Magnetic Stimulation, Paired Pulse” or “Transcranial Magnetic Stimulation, Repetitive” or “Non-invasive electrical stimulation”)  Indexes=SCI-EXPANDED, SSCI, A&HCI, CPCI-S, CPCI-SSH, BKCI-S, BKCI-SSH, ESCI, CCR-EXPANDED, IC Timespan=All years |  |  |
| # 2 | TOPIC: ("Stroke" or "Cerebrovascular Accident" or "Brain Vascular Accident" or "Apoplexy" or "Cerebral Stroke" or "Cerebrovascular Apoplexy")  Indexes=SCI-EXPANDED, SSCI, A&HCI, CPCI-S, CPCI-SSH, BKCI-S, BKCI-SSH, ESCI, CCR-EXPANDED, IC Timespan=All years |  |  |
| # 1 | TOPIC: ("Aphasia" or "Alogia" or "Logasthenia" or "Word Deafness" or "Anepia" or "Aphasia, Semantic" or "Aphasia, Syntactical" or "Aphasia, Progressive" or "Aphasia, Acquired" or "Aphasia, Ageusic" or "Aphasia, Auditory Discriminatory" or "Aphasia, Commisural")  Indexes=SCI-EXPANDED, SSCI, A&HCI, CPCI-S, CPCI-SSH, BKCI-S, BKCI-SSH, ESCI, CCR-EXPANDED, IC Timespan=All years |  |  |
